# Molecular Dissection of Neurobeachin Function at Excitatory Synapses

**DOI:** 10.3389/fnsyn.2018.00028

**Published:** 2018-08-15

**Authors:** Daniele Repetto, Johannes Brockhaus, Hong J. Rhee, Chungku Lee, Manfred W. Kilimann, Jeongseop Rhee, Lisa M. Northoff, Wenjia Guo, Carsten Reissner, Markus Missler

**Affiliations:** ^1^Institute of Anatomy and Molecular Neurobiology, Westfälische Wilhelms-University, Münster, Germany; ^2^Synaptic Physiology Group, Max-Planck Institute for Experimental Medicine, Göttingen, Germany

**Keywords:** synaptic transmission, dendritic spine, autism, AMPA receptor, neurobeachin, hippocampus

## Abstract

Spines are small protrusions from dendrites where most excitatory synapses reside. Changes in number, shape, and size of dendritic spines often reflect changes of neural activity in entire circuits or at individual synapses, making spines key structures of synaptic plasticity. Neurobeachin is a multidomain protein with roles in spine formation, postsynaptic neurotransmitter receptor targeting and actin distribution. However, the contributions of individual domains of Neurobeachin to these functions is poorly understood. Here, we used mostly live cell imaging and patch-clamp electrophysiology to monitor morphology and function of spinous synapses in primary hippocampal neurons. We demonstrate that a recombinant full-length Neurobeachin from humans can restore mushroom spine density and excitatory postsynaptic currents in neurons of Neurobeachin-deficient mice. We then probed the role of individual domains of Neurobeachin by comparing them to the full-length molecule in rescue experiments of knockout neurons. We show that the combined PH-BEACH domain complex is highly localized in spine heads, and that it is sufficient to restore normal spine density and surface targeting of postsynaptic AMPA receptors. In addition, we report that the Armadillo domain facilitates the formation of filopodia, long dendritic protrusions which often precede the development of mature spines, whereas the PKA-binding site appears as a negative regulator of filopodial extension. Thus, our results indicate that individual domains of Neurobeachin sustain important and specific roles in the regulation of spinous synapses. Since heterozygous mutations in Neurobeachin occur in autistic patients, the results will also improve our understanding of pathomechanism in neuropsychiatric disorders associated with impairments of spine function.

## Introduction

Dendritic spines (DSs) are small, actin-enriched protrusions that arise from neuronal dendrites and receive most of the excitatory input in brain circuitries (Hering and Sheng, 2001; Hotulainen and Hoogenraad, 2010; Segal, 2010). DS play a pivotal role during synaptic transmission because of their ability to compartmentalize biochemical signaling and integrate presynaptic input (Bourne and Harris, 2008; Cingolani and Goda, 2008; Yuste, 2011). To accomplish this, DS undergo adaptive changes during development, in response to sensory stimuli or during cognitive processes such as learning and memory (Bhatt et al., 2009; Holtmaat and Svoboda, 2009; Lin and Koleske, 2010; Berry and Nedivi, 2017). Mature spines typically display a mushroom-shaped morphology with a well-distinguishable head and neck that contain the postsynaptic signaling machinery (Harris and Weinberg, 2012). Accordingly, numerous studies revealed that spine morphology and their function are mutually dependent (Kasai et al., 2010; Sala and Segal, 2014; Moyer and Zuo, 2018). In addition, important evidence has linked several neuropsychiatric disorders and behavioral deficits to the impairment of different spine types (Lin and Koleske, 2010; Penzes et al., 2011; Joensuu et al., 2018).

We and other groups have previously addressed the function of the neuronal BEACH (beige and Chediak-Higashi) protein Neurobeachin (Nbea), and observed that Nbea is involved in DS formation, synaptogenesis, and synaptic transmission in different species such as mouse (Su et al., 2004; Medrihan et al., 2009; Niesmann et al., 2011; Nair et al., 2013), zebrafish (Miller et al., 2015), and Drosophila (Volders et al., 2012; Wise et al., 2015). Mammalian Nbea is a large (327 kDa), cytosolic multidomain protein peripherally associated with neuronal membranes (Wang et al., 2000), and concentrated at the *trans*-Golgi network, post-Golgi vesicles, and at synaptic contacts (Wang et al., 2000; Niesmann et al., 2011; Volders et al., 2012; Nair et al., 2013). This localization is reflected by the morphological and functional defects found at synapses throughout the central and peripheral nervous system of Nbea knockout (KO) mice (Su et al., 2004; Medrihan et al., 2009; Niesmann et al., 2011; Nair et al., 2013). It is also consistent with the idea that Nbea regulates trafficking of glutamate and GABA_A_ receptors to postsynaptic sites (Nair et al., 2013; Farzana et al., 2016).

Nbea contains at least seven distinct protein motifs or domains (Cullinane et al., 2013): First, an A-kinase anchoring protein (AKAP) motif that binds to PKA (Wang et al., 2000); second, a conserved BEACH domain that is in tight complex with, third, a Pleckstrin-Homology domain (PH) (Jogl et al., 2002; Gebauer et al., 2004); fourth, an Armadillo repeat domain originally proposed as “domain-of-unknown-function” (DUF4704); fifth, a Concanavalin A-like lectin domain (Burgess et al., 2009); sixth, another DUF domain (DUF1088) with a putative nuclear localization signal (Tuand et al., 2016); and finally, four C-terminal tryptophan-aspartic acid (WD 40) repeats. A binding study with recombinant mouse Nbea showed that the c-terminus including DUF1088-PH-BEACH-WD40 binds to SAP102. A single point mutation that disrupts the tight complex of PH and BEACH domain in full-length Nbea prevents SAP102 binding, although the isolated PH-BEACH domain does not bind to SAP102 (Lauks et al., 2012). Moreover, since human and mouse Nbea share 98% identical residues with some domains reaching 100% (Lectin, AKAP motif, and WD40 repeats), we hypothesized that it is possible to analyze human disease related single site mutations in neuronal cultures from mice. This high homology also allows to use detailed structural data of the PH-BEACH domain derived from human Nbea (Jogl et al., 2002).

Several reports have emphasized the medical importance of Nbea in humans. Nbea spans a common fragile site on human chromosome 13q13 that was found disrupted by translocation in idiopathic cases of non-familial autism (Castermans et al., 2003, 2010). In support, Nbea haploinsufficiency in mice recapitulates the human disorder including autism-like behaviors and social deficits (Nuytens et al., 2013). In addition, there are mutations in closely related proteins, for example in NBEAL2 that cause platelet abnormalities with impaired secretory α-granule biogenesis in patients (Albers et al., 2011; Gunay-Aygun et al., 2011; Kahr et al., 2011), and in LRBA which are linked to immunological deficit and autoimmunity (Lopez-Herrera et al., 2012). In an effort to address putative pathomechanisms, we have therefore investigated the contribution of recombinant full-length human Nbea and its individual domains to synaptic function in our present study. Specifically, we tested the ability of human Nbea domains to rescue the functional and morphological defects apparent at synapses of primary hippocampal neurons from Nbea KO mice. We show that expression of PH-BEACH domains of Nbea is sufficient to restore normal DS density and surface targeting of AMPAR subunits. In addition, we identify a cooperation of the Armadillo domain and PKA binding motif in regulating filopodia extension in opposite directions. Together, our data provide the first insights into specific roles of individual Nbea domains in synapse function.

## Materials and Methods

### Animals

Wild-type and Nbea KO mutant mice of either sex were used for neuronal cultures derived from timed-pregnant dams at E17. Animal experiments were performed at the University of Münster in accordance with government regulations for animal welfare and approved by the Landesamt für Natur, Umwelt und Verbraucherschutz (LANUV, NRW, Germany), license numbers 84-02.05.20.11.209 and 84-02.04.2015.A423.

### Cell Culture

Dissociated primary neurons were prepared in Hank’s Balanced Salt Solution (HBSS) from hippocampi as described (Neupert et al., 2015). Briefly, cell suspensions obtained after 0.25% trypsin and trituration were plated onto 18 mm glass coverslips (Menzel-Glaeser, Braunschweig, Germany) coated with poly-L-lysine (Sigma) at a density of 55,000 cells/coverslip. After 4 h at 37°C in plating medium (MEM, 10% horse serum, 0.6% glucose, 1 mM sodium pyruvate), coverslips were inverted onto a 70–80% confluent monolayer of astrocytes grown in 12-well plates (Falcon), and incubated in Neurobasal medium supplemented with B27, 0.5 mM glutamine and 12.5 μM glutamate. After 3 days, media were refreshed with Neurobasal medium supplemented with B27, 0.5 mM glutamine and 5 μM AraC. Cultures were maintained at 37°C in a humidified incubator with an atmosphere of 95% air and 5% CO_2_. Neurons were transfected at days *in vitro* (DIV) 14–16 using lipofectamine (Thermo Fisher Scientific, Waltham, MA, United States), and experiments performed between DIV 17–21. Since endogenous Nbea is highly expressed during the period of intense DS formation before synapse maturation is fully established (Nwabuisi-Heath et al., 2012; Nair et al., 2013), our transfection strategy was actually designed to fit to the time course of expression of the endogenous protein.

Autaptic cultures of hippocampal neurons were prepared on micro islands of astrocytes as described previously (Burgalossi et al., 2012). In brief, astrocytes were obtained from mouse cortices from P0 WT animals using digestion with 0.25% trypsin (Gibco) for 20 min at 37°C. The cells were plated in T75 culture flasks in DMEM medium (Gibco) containing 10% FBS (PAA) and penicillin/streptomycin (Gibco). The medium was exchanged the day after plating, and cells were allowed to grow for 7–10 days. Following this, cells were collected from the flask using trypsin digestion and plated at a density of 12,000 cells/well on 32 mm coverslips. The coverslips used for micro island cultures were first coated with agarose (Sigma-Aldrich), and then with a coating solution containing poly-D-lysine (Sigma-Aldrich), acetic acid, and collagen (BD), using a custom-made stamp to generate 400 μm × 400 μm substrate islands. Hippocampi from embryonic day 18 (E18) mouse embryos were dissected free of meninges and separately collected in ice-cold Hanks Buffered Salt Solution (HBSS; Gibco). They were incubated in papain solution containing 2 mg cysteine, 10 ml DMEM (Gibco), 1 mM CaCl_2_, and 0.5 mM EDTA, along with 20–25 units of papain (Worthington Biomedical Corporation) for 45 min for at 37°C. After washing, cells were triturated and counted in a Fuchs-Rosenthal or Neubauer chamber. The cells were plated in pre-warmed Neurobasal medium (Gibco) supplemented with B-27 (Gibco), glutamax (Gibco) and penicillin/streptomycin (Gibco) at a density of 4,000 cells/well on a 32 mm coverslip for micro island cultures.

### Dendritic Spine Analysis

Dendritic spines were analyzed from cytosolic marker t-dimer-RFP transfected neurons. RFP-positive neurons were imaged with a confocal spinning disk Axio Observer-Z1 (Visitron) with an EMCCD camera (ImagEM 512 CCD, Hamamatsu), using 63x Plan-Neofluar oil immersion objectives. 0.5 μm Z-stacks were acquired, with a maximal distance of 1.5 μm from the focus plane. All images related to GFP and 647 nm channels were acquired with the same laser power, exposure time and camera gain settings. Using ImageJ software, maximum intensity projections of Z-stacks were generated for the analysis, and mushrooms spines and filopodia protrusions quantified on all first and second order dendrites for each imaged cell. The total length of these dendrites was measured and all mushroom and filopodia protrusions were counted to obtain the number of protrusion/20 μm dendritic length (Repetto et al., 2014). Data for number of protrusions/20 μm per cell are based on about 350–500 protrusions for each neuron. For the quantification of Nbea domains in the spine head, regions of interests (ROIs) were manually drawn with ImageJ software around at least 50 spine heads per neuron. In this experiment, only mushroom spines with clearly visible heads were selected randomly from the first and second order dendrites. After background subtraction, the ROIs series was copied over the fluorescent images correspondent to the GFP signal to obtain a fluorescence intensity value of GFP fused domain in DS head. In addition, for the quantification of the individual Nbea domains in dendritic shafts, 20 μm long dendritic windows excluding the spines were manually delineated on t-dimer-RFP transfected neurons. At least three first order and three second order dendrites were randomly selected for each neuron.

For FRAP experiments, WT and Nbea KO hippocampal neurons were transfected at DIV 14 with EGFP-Actin, cloned under the control of the human synapsin promoter. Transfected cells were imaged with the spinning disk confocal setup described above. The spine heads were bleached with a laser beam at 20% power and images were acquired every 2 s to measure EGFP-actin recovery. To improve the reliability of measurements, each spine was imaged twice with a recovery interval of at least 5 min. After background subtraction, fluorescence intensity in DS was measured for each time point. The series of fluorescence intensity values was fitted to a first order exponential equation using the FRAP profiler plug-in available in ImageJ software. After the exponential curve fitting, the halftime recovery of EGFP-Actin in spine head, indicating actin turnover rate was extracted and analyzed.

To monitor the dynamics of spines under basal conditions, WT and Nbea KO hippocampal neurons were transfected at DIV 14 with GFP or Life-Actin fused to RFP (Riedl et al., 2008). Live imaging confocal microscopy of DS was performed with the same confocal setup described above and acquiring up to six Z-stacks every 15 s for 30 min. Single spines in each time point were analyzed extracting the coordinates of the center of mass (COM) using ImageJ software and the particles analysis tool. The derivative in respect of time for the *x* and *y* coordinates values was calculated and the absolute value was derived. The sum of the absolute values for *x* and *y* coordinates, indicating the COM displacement was calculated and used for statistical analysis.

### Immunocytochemistry and GluA2 Surface Staining

For surface staining of GluA2 subunit of AMPA receptor the experiment was carried out as reported (Aoto et al., 2013). Briefly, live DIV 21 neurons were washed quickly in PBS supplemented with 0.5 mM CaCl_2_, 1 mM MgCl_2_ and 4% sucrose (PBS-MC). Monoclonal mouse anti-GluA2 antibody (Millipore MAB397) against an extracellular epitope of GluA2 AMPA receptor subunit was incubated for 10 min at 37°C in incubator. After a brief wash in cold PBS-MC, cells were fixed with 4% paraformaldehyde/4% sucrose for 15 min and blocked in detergent free 10% normal goat serum PBS solution. To analyze the surface GluA2 fluorescence intensity, rectangular windows of at least 20 μm length were manually drawn around RFP transfected dendrites. These regions were copied to the corresponding fluorescent image of the anti-GluA2 labeling (Alexa 647 goat-anti-mouse IgG) and, after background subtraction, an intensity value was obtained.

For immunocytochemistry of hippocampal cultures, DIV 21 neurons were fixed with 4% paraformaldehyde/4% sucrose for 8 min, washed with PBS, blocked in 10% normal goat serum (NGS), 0.1% Triton-X100/PBS for 1 h, and incubated with Alexa Fluor Phalloidin 633 (Thermo Fisher Scientific) staining F-Actin for 1 h at RT. After additional washings in PBS, coverslips were embedded in mounting medium (Dako).

### Expression Vectors

We used a mammalian expression vector containing the human synapsin 1 promoter and inserted EGFP to create hSYN1-EGFP-C. To generate GFP-Nbea (Nbea_Human, Q8NFP9), we introduced MluI and NotI sites to flank CMV-YFP in human YFP-Nbea (IMAGE clone 100069291) using quikchange primers (MM11-M, 5′-GCC ATG CAT TAG TTA TTA ACG CGT TAG TAA TCA ATT ACG GGG-3′, +MluI; MM11-N, 5′-GTA CAA ACT TGT TGA TGA TCG CGG CCG CGA CTT GTA CAG CTC GTC CAT GCC G-3′, +NotI). The MluI/NotI fragment was replaced by a correspondent PCR amplicon containing hSYN1-EGFP amplified from hSYN1-EGFP-C using primers (MM11-131, 5′-TTA TTA ACG CGT CTA GAC TGC AGA GGG CCC TGC GTA TGA GT-3′, +MluI; MM11-132, 5′-GAT GAT CGC GGC CGC GAC TTG TAC AGC TCG TCC ATG CCG AGA GTG-3′, +NotI) resulting in hSYN1-EGFP-Nbea. An EcoRI fragment of GFP-Nbea was subcloned into pbluescript to delete coding region of PKA site 1091-EVESLLDNVYSAAVEKLQN-1109 using quikchange primer (MM12-45, 5′-GGT GGA GAG AAT GGT GCA CTA GTG AAT GTA CAT GGA AGT GTT GG-3′, +SpeI) and re-ligated to obtain GFP-NbeaΔPKA. Single N-terminal EGFP-fused Nbea domains were obtained by PCR using following primers for lectin 231-TFN…STF-416 (MM12-68, 5′-GCC TGC AGG TCG ACA CTT TTT TCA ATT TCC CTG GTT GTA GCG C-3′, +SalI; MM12-69, 5′-GGA TCC TCT AGA GAA GGT ACT CTT ATA TCC AGG TCC TAA CTG ATG AAT TGC-3′, +XbaI), ARM-long 505-LFA…QEE-1156 (MM12-47, 5′-GCC TGC AGG TCG ACC TTT TTG CCC AAT TGG ATA ATA GGC AGC TCA ATG-3′, +SalI; MM12-48, 5′-ATC CTC TAG ATT CCT CCT GTT TGG GTA TTT TAT CAA AGA GAA ATG-3′, +XbaI), DUF1088 1966-EGR…EGD-2133 (MM12-84, 5′-CTG CAG GTC GAC GAA GGA AGA TTA CTG TGC CAT GCT ATG AAG-3′, +SalI; MM12-85, 5′-GAT CCT CTA GAG TCT CCT TCC AGC ATA AGT TCT GTC TC-3′, +XbaI), PH-BEACH 2150-GPV…PPR-2563 (MM12-70, 5′-GCC TGC AGG TCG ACG GCC CAG TGG TTC TCA GCA CCC CTG-3′, +SalI; MM12-71, 5′-GGA TCC TCT AGA CCG AGG CGG ATG TGG CTC AAT AAG CAA CTG-3′, +XbaI), WD40 2718-GHW…HYE-2941 (MM13-01, 5′-CTG CAG GTC GAC GGC CAT TGG GAT GTG GTC ACT TG-3′, +SalI; MM13-02, GAT CCT CTA GAC TCA TAA TGC CAC CGA TTA AAA TCT ATA TTA AAA GC-3′, +XbaI) and insertion into hSYN1-EGFP-C. Arm-coreΔPKA was generated by introducing two STOP signals after Arm-core domain 505-LFA…YHA-423 in Arm-long using quikchange primer (MM16-13, 5′-CAA GAG GAG GAA AAC ATA TGA TAA AAA AAG GGA AAG AAA GGG-3′, +NdeI). All mutations were performed by Quickchange method (Agilent) using shown forward and correspondent reverse complement primer including named silent restriction site. All constructs were validated by sequencing (GATC, Konstanz).

### Electrophysiological Recordings

For patch clamp recordings, coverslips with cultured neurons (DIV 17–20) were placed in a recording chamber mounted to an inverted microscope (Observer.A1, Zeiss, Oberkochen, Germany) and superfused at 1.0–1.5 ml/min with bath solution at room temperature (≈21°C), containing (in mM): NaCl 145, KCl 3, MgCl_2_ 1.5, CaCl_2_ 1.5, glucose 11, HEPES 10; pH 7.4 adjusted with NaOH; to isolate excitatory miniature postsynaptic currents (mEPSCs), 10 μM bicuculline and 0.5 μM tetrodotoxin were added. Patch pipettes (borosilicate glass, 1.5 mm outer diameter; Hilgenberg, Malsfeld, Germany) were pulled by a two-stage electrode puller (PIP 6, HEKA Elektronik, Lambrecht, Germany), showing resistances of 2–3 MΩ when filled with pipette solution containing (in mM): 140 K-gluconate, 1 CaCl_2_, 2 MgCl_2_, 4 Na-ATP, 0.5 Na-GTP, 10 EGTA, 10 Hepes, pH 7.3. Whole-cell currents were recorded with an EPC 10 USB Double patch-clamp amplifier and Patchmaster software (HEKA Elektronik). Signals were filtered at 3 kHz and digitized at 10 kHz, series resistance was compensated by about 60–80%. Cells were held at -70 mV in whole-cell configuration, series resistance and membrane capacitance compensated. For evaluation of EPSCs, more than 100 consecutive EPSCs per cell from continuous recordings (100 s) were chosen and analyzed with Minianalysis software (Synaptosoft, Decatur, GA, United States).

Autaptic neurons (11–14 DIV) were whole-cell voltage clamped at -70 mV with an Axoclamp amplifier under the control of the Clampex program 10.1. All analyses were performed using Axograph X. The experiments were performed using a patch-pipette solution containing (in mM) 136 KCl, 17.8 Hepes, 1 EGTA, 0.6 MgCl_2_, 4 NaATP, 0.3 mM Na_2_GTP, 15 creatine phosphate, and 5 U/mL phosphocreatine kinase (315–320 mOsmol/L, pH 7.4). The extracellular solution used for all recordings contained (in mM) 140 NaCl, 2.4 KCl, 10 Hepes, 10 glucose, 4 CaCl_2_ and 4 MgCl_2_ (320 mOsml/liter), pH 7.3. Evoked EPSCs were induced by depolarizing the cell from -70 to 0 mV at a frequency of 0.2 Hz. Short-term plasticity was evaluated by recording PSCs during a 10 Hz stimulation train. The series resistance was compensated by about 60–80%.

### Statistical Analysis

No statistical methods were engaged to predetermine sample size, instead we based our experimental design on numbers reported in previous studies (Niesmann et al., 2011; Nair et al., 2013). Statistical tests were performed either with GraphPad Prism (GraphPad Software, La Jolla, CA, United States) or Excel (Microsoft). If samples met criteria for normality, we used a Student’s *t*-test to compare two groups, and a one-sided ANOVA for more than two groups. If ANOVAs were significant, we used a *post hoc* Tukey’s multiple comparisons test to compare groups. Data are presented as means ± SEM. Significance levels were as indicated in figures: ^∗^*P* < 0.05; ^∗∗^*P* < 0.01; and ^∗∗∗^*P* < 0.001.

### Structural Modeling

Neurobeachin is a multidomain protein with largely unknown molecular fold. The crystal structure of the PH and BEACH domain complex has been solved (PDB: 1MI1) and a model for the lectin-like domain is available (Burgess et al., 2009). We have used the coordinates described in that study (PDB_ID: 1AF9) to model the lectin-like domain. A common 7-bladed beta propeller (PDB_ID: 2CE8 and 1K8K) served as template for Nbea WD40 repeats. We used threading method phyre2 (Kelley et al., 2015) to generate a model of an armadillo domain of high confidence (>97%) that was built mainly from importin-α-1 coordinates as template (PDB_ID:2JDQ). Using the Arm-long sequence which includes the AKAP motif, phyre2 gave the same Arm-core but with an extended helical domain and an AKAP helix attached to the Arm-core. The remainders, including DUF1088, are domains with low structural complexity. We used phyre2 to generate compact space-filling models to complete the full-length structure of Nbea.

## Results

### Full-Length Nbea Restores Synaptic Transmission in Knockout Neurons

Homozygous Nbea KO mice die after birth by asphyxiation due to defects of synaptic transmission at neuromuscular junctions and synapses in the respiratory network (Su et al., 2004; Medrihan et al., 2009). Here, we analyzed the role of Nbea by performing rescue experiments in primary hippocampal neurons cultured from individual fetal KO mice. We focused our analysis on excitatory synapses because of emerging evidence that interaction partners and signaling pathways of Nbea may differ at inhibitory contacts (del Pino et al., 2011; Farzana et al., 2016).

**FIGURE 1:**
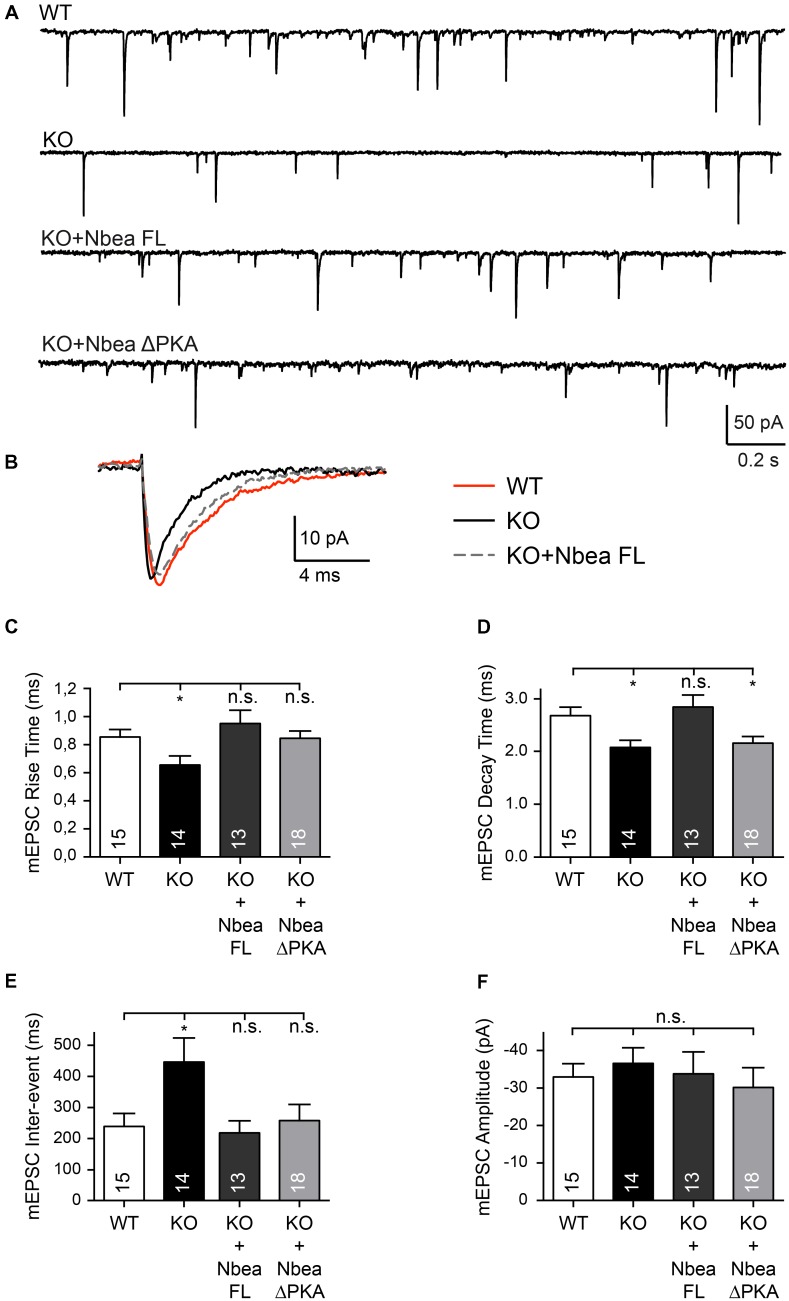
Full-length human Nbea restores EPSC defects in null-mutant neurons. **(A)** Representative traces of continuous recordings of mEPSCs from cultured hippocampal neurons of WT, Nbea KO (KO), KO transfected with human full-length Nbea (KO + Nbea FL) and KO transfected with NbeaΔPKA (KO + NbeaΔPKA). **(B)** Averaged traces of mEPSCs recorded from WT (red line), KO (black), and KO + Nbea FL (dashed) neurons indicate faster kinetics in Nbea KO that can be rescued by Nbea FL. **(C–F)** Quantitative analysis of rise time **(C)**, decay time **(D)**, inter-event interval **(E)** and amplitude **(F)** of mEPSCs in WT, KO, KO + Nbea FL and KO + NbeaΔPKA. Digits in bars give the number of recorded neurons; from each neuron more than 100 consecutive mEPSCs were analyzed for evaluation. Data are means ± SEM; ^∗^*P* < 0.05, n.s., not significant; by one-way ANOVA with Tukey’s multiple comparisons test.

To probe the ability of a newly constructed full-length human Nbea (Nbea FL) to rescue major functional impairments of excitatory KO synapses observed earlier (Medrihan et al., 2009; Niesmann et al., 2011; Nair et al., 2013), we first recorded miniature excitatory postsynaptic currents (mEPSC) from neuronal cultures of Nbea KO and control animals in presence of TTX and bicuculline (**Figures 1A–F**). Consistent with the previous reports, Nbea KO neurons showed a faster mEPSC rise time (WT: 0.85 ± 0.05 ms, *N* = 15; KO: 0.67 ± 0.05, *N* = 14; *P* = 0.045) (**Figure 1C**) and a faster decay time (WT: 2.68 ± 0.16 ms, *N* = 15; KO: 2.079 ± 0.14, *N* = 14; *P* = 0.01) (**Figure 1D**) that are in agreement with the shift from mature spinous to shaft synapses when Nbea is deleted because a faster kinetic could be caused by lack of dendritic filtering in thin spine necks (Araya et al., 2006; Niesmann et al., 2011). A significantly longer interevent interval time of Nbea KO mEPSCs compared to WT was also observed in our experiments (WT: 241.2 ± 44.3 ms, *N* = 15; KO: 451.1 ± 78.5, *N* = 14; *P* = 0.012) (**Figure 1E**), whereas we did not see significant differences in the amplitude of mEPSCs (WT: -32.75 ± 3.9 pA, *N* = 15; KO: -36.53 ± 4.2, *N* = 14, *P* = 0.995) (**Figure 1F**). Strikingly, transfection of a human EGFP-tagged full-length Nbea construct into KO neurons was able to rescue completely the defects in rise time (KO + NbeaFL: 0.95 ± 0.09 ms, *N* = 13; *P*_WT_ = 0.31; *P*_KO_ = 0.004), decay time (KO + NbeaFL: 2.85 ± 0.22 ms, *N* = 13; *P*_WT_ = 0.48; *P*_KO_ = 0.001) and interevent intervals (KO + NbeaFL: 221.8 ± 39.18 ms, *N* = 13; *P*_WT_ = 0.82; *P*_KO_ = 0.008) of mEPSCs (**Figures 1C–E**). These data suggest that the phenotype was specific for the Nbea deletion and was not caused by compensatory mechanisms.

**FIGURE 2:**
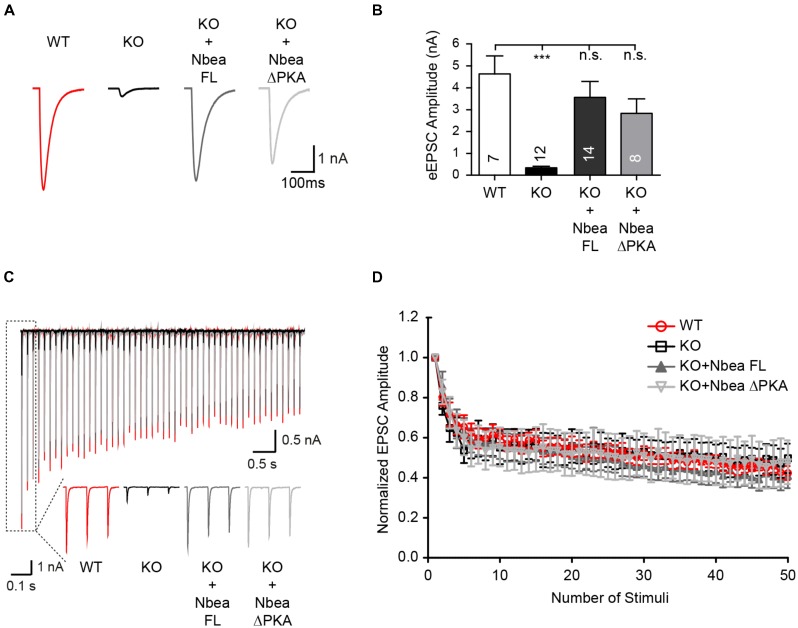
Evoked EPSCs at autaptic synapses are restored by full-length Nbea and NbeaΔPKA. **(A)** Sample traces of evoked EPSCs recorded from individual hippocampal neurons grown on micro islands that form autapses. The eEPSCs shown are from untransfected WT, from Nbea KO neurons mock transfected with GFP alone, and transfected with Nbea FL or NbeaΔPKA constructs. **(B)** Average values of eEPSC amplitudes from WT, KO/GFP, Nbea FL, and NbeaΔPKA. Data are means ± SEM; ^∗∗∗^*P* < 0.001, n.s., not significant; by one-way ANOVA with Tukey’s multiple comparisons test. **(C)** Overlay of representative traces of eEPSCs during AP stimulation trains (10 Hz) from WT, KO/GFP, KO + Nbea FL and KO + NbeaΔPKA; insets depict magnifications of the response to the first three stimuli. **(D)** Change in EPSC amplitudes during a 10 Hz stimulation train in WT, KO, KO + Nbea FL and KO + NbeaΔPKA autaptic glutamatergic neurons. N_WT_ = 5 neurons, N_KO_ = 7 neurons, N_KO+NbeaFL_ = 11 neurons, N_KO+NbeaΔPKA_ = 6 neurons.

Previous analyses of Nbea-deficient neurons found that in contrast to spontaneous release events (**Figure 1F**), the amplitudes of evoked postsynaptic responses from excitatory synapses are affected more strongly by the lack of Nbea, reflecting the impaired surface targeting of AMPAR subunits that limits the number of available postsynaptic receptors (Nair et al., 2013). While a smaller amount of glutamate receptors in Nbea KO neurons may be enough to respond to the release by single vesicles with normal amplitudes, the response to action potential induced glutamate release is more than saturating the reduced number of postsynaptic receptors in Nbea KO neurons. Here, we confirmed the dramatic reduction of evoked EPSCs (eEPSC) in autaptic hippocampal neurons (**Figure 2A**), a model system in which single neurons grow on top of small glia cell islands to allow superior analysis of pre- and postsynaptic properties of synaptic transmission (Rosenmund et al., 1993; Burgalossi et al., 2012). The eEPSC amplitudes of Nbea KO neurons, mock-transfected with GFP as control, was strikingly smaller than those of wild-type neurons (WT: 4.63 ± 0.82 nA, *N* = 7; KO: 0.33 ± 0.068, *P* = 0.0003; *N* = 12) (**Figure 2B**). We now validated that this is a specific effect by expressing the full-length Nbea in KO neurons, which was able to restore eEPSC amplitudes back to normal levels (KO + NbeaFL: 3.56 ± 0.71 nA, *N* = 12; *P*_WT_ = 0.65; *P*_KO_ = 0.001). Together, these results demonstrate that expression of a full-length human Nbea in KO neurons restores the defects in both spontaneous and action potential-driven excitatory transmission. In addition, to screen for putative presynaptic defects that might depend on Nbea deletion, short-term plasticity was analyzed in the hippocampal autaptic cultures. eEPSC amplitudes in KO, KO transfected with Nbea FL and KO transfected with NbeaΔPKA depressed progressively during 10 Hz stimulation trains to a comparable steady-state depression level of about 50% as WT control neurons (**Figures 2C,D**). Moreover, comparing the first and second responses of these stimulation series, no significant differences in paired-pulse ratio were observed (WT: 0.80 ± 0.04, *N* = 5; KO: 0.76 ± 0.05, *N* = 7; KO + NbeaFL: 0.86 ± 0.11, *N* = 11; KO + NbeaΔPKA: 0.83 ± 0.07, *N* = 6). Thus, in extension of a previous study that emphasized more a presynaptic role of Nbea at synapses of the brainstem (Medrihan et al., 2009), our current data confirm the preponderance of postsynaptic defects in hippocampal neurons in absence of Nbea (Nair et al., 2013).

A successful rescue analysis as shown above not only attests to the specificity of the phenotype observed in null mutant neurons but opens the possibility to dissect the molecular basis of the process. Nbea’s PKA binding site probably contributes to its function, and rescue experiments have been carried out (Farzana et al., 2016) by deleting the 19 residues long putative AKAP motif (Wang et al., 2000) in recombinant Nbea. Experiments with exogenously applied neurotransmitters suggested that the AKAP motif of Nbea is required for the regulation of GABA-induced postsynaptic responses but not for glutamate responses (Farzana et al., 2016). To probe the role of the PKA binding site in spontaneous and evoked transmission in our excitatory hippocampal neurons, we deleted the motif in the human Nbea construct (NbeaΔPKA; lacking residues 1091–1109: EVESLLDNVYSAAVEKLQN) and repeated the rescue experiments. The NbeaΔPKA mutant was expressed at levels comparable to the full-length Nbea in our hippocampal neurons as predicted from previous reports (Lauks et al., 2012; Farzana et al., 2016). Nbea lacking the PKA binding site was able to fully restore the increased interevent interval time of KO mEPSCs (NbeaΔPKA: 260 ± 53 ms, *N* = 18, *P*_WT_ = 0.81; *P*_KO_ = 0.017; **Figure 1E**), and almost completely rescued the shorter mEPSC rise time (NbeaΔPKA: 0.84 ± 0.05 ms, *N* = 18, *P*_WT_ = 0.91; *P*_KO_ = 0.047; **Figure 1E**) and the reduced amplitude of eEPSCs (NbeaΔPKA: 2.83 ± 0.66 nA, *N* = 8, *P*_WT_ = 0.32; *P*_KO_ = 0.045; **Figures 2A,B**). Although it remains unclear why the NbeaΔPKA construct was less effective on the decay time of mEPSCs (NbeaΔPKA: 2.17 ± 0.13 ms, *N* = 18, *P*_WT_ = 0.022; *P*_KO_ = 0.69), it emerges from these data that the PKA binding site is not critically required for the role of Nbea in excitatory synaptic transmission.

### PH-BEACH Domain Affects Synaptic Targeting of AMPA Receptors and Controls Spine Density

**FIGURE 3:**
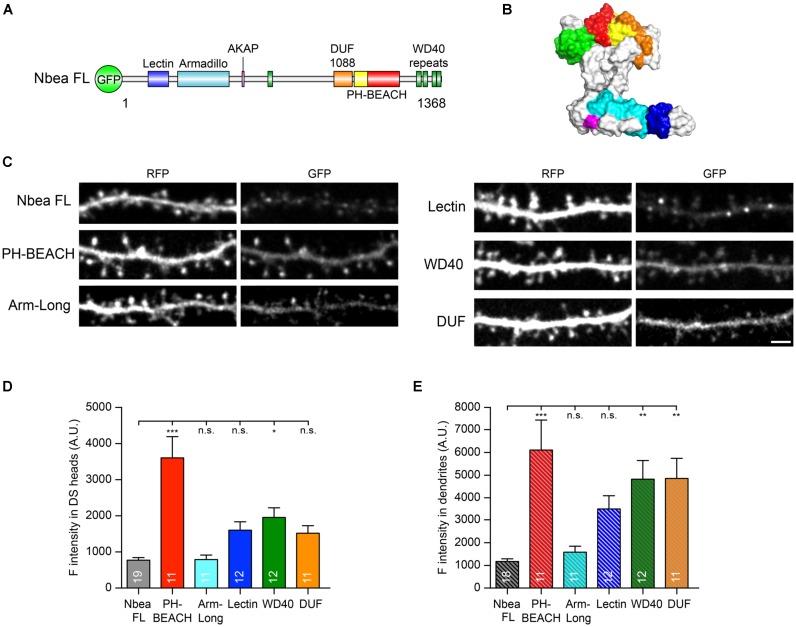
Individual Nbea domains are able to localize to dendrites and spines. **(A)** Nbea motif architecture depicting its multidomain composition. GFP, N-terminally fused EGFP tag; Lectin, concanavalin A-like lectin domain; Armadillo, armadillo repeat; AKAP, A-kinase anchoring protein; DUF1088, domain-of-unknown-function; PH, pleckstrin homology-like domain; BEACH, *Be*ige *a*nd *C*hediak-*H*igashi domain; WD40, tryptophan-aspartic acid repeats. **(B)** Predicted structural model of Nbea with domains color coded as in **(A)**. See “Materials and Methods” section for details on templates and modeling procedure. **(C)** Representative images of dendrites from primary hippocampal neurons transfected with cytosolic marker t-dimer-RFP and isolated GFP-tagged Nbea domains as indicated. Scale bar: 2.5 μm. **(D)** Quantification of fluorescence intensity of individual Nbea domains in dendritic spine (DS) heads. Data are means ± SEM (arbitrary units, A.U.). *N*, number of neurons (in bars); quantification was carried out on at least 50 spines per neuron. One-way ANOVA with Tukey’s multiple comparisons test; n.s., not significant, ^∗^*P* < 0.05, ^∗∗∗^*P* < 0.001. **(E)** Quantification of fluorescence intensity of individual Nbea domains in dendritic shaft. Data are means ± SEM (arbitrary units, A.U.). *N*, number of neurons (in bars); quantification was carried out on at least four 20 μm-long dendritic windows per neuron. One-way ANOVA with Tukey’s multiple comparisons test; n.s., not significant, ^∗∗^*P* < 0.01, ^∗∗∗^*P* < 0.001.

The amino acid sequence of Nbea includes a Concanavalin A-like lectin domain (Lectin, blue in **Figures 3A,B**) near the N-terminus, followed by an Armadillo repeat (Armadillo, turquoise in **Figures 3A,B**), the AKAP motif addressed above (PKA binding site, magenta in **Figures 3A,B**), a “domain-of-unknown-function” (DUF1088, orange in **Figures 3A,B**), the namesake complex of PH-BEACH domains (PH-BEACH, yellow and red in **Figures 3A,B**) and C-terminal WD40 repeats (WD, green in **Figures 3A,B**). For rescue experiments using these individual Nbea domains, we focused on the lower postsynaptic AMPA receptor availability (Nair et al., 2013) and the reduced number of mature DSs (Niesmann et al., 2011) in Nbea KO neurons. As a prerequisite for such assays, we asked if and how efficiently individual domains were targeted to the postsynaptic compartment of DSs in comparison to full-length Nbea.

To investigate the subcellular targeting, we generated GFP-tagged expression constructs under the control of human synapsin 1 promoter, analogous to the full-length Nbea construct used in the electrophysiological experiments above (**Figures 1**, **2**). Nbea KO neurons were co-transfected with GFP-fused Nbea domain constructs and a soluble cytosolic marker to identify DS morphology, t-dimer-RFP. Interestingly, all individual domains reached dendritic branches and spines (**Figure 3C**) and showed at least the expression levels of full-length Nbea (**Figure 3D**). The Armadillo domain (Arm-long in **Figure 3C**) showed a fluorescence intensity in spine heads that was almost identical to full-length Nbea (Nbea FL: 784.3 ± 65.3 A.U., *N* = 19; Arm-long: 788.7 ± 126.7, *N* = 11, *P*_NbeaFL_ = 0.99; **Figure 3D**). Lectin, WD40 and DUF domains showed a tendency of higher fluorescence intensity in spine heads compared to full-length Nbea but only WD40 reached a significant difference (Lectin: 1,604 ± 230, *N* = 12, *P*_NbeaFL_ = 0.19; WD40: 1,955 ± 268, *N* = 12 *P*_NbeaFL_ = 0.012; DUF: 1,519 ± 208, *N* = 11 *P*_NbeaFL_ = 0.34; **Figure 3D**). Importantly, transfection of the PH-BEACH domain of Nbea resulted in the highest fluorescence intensity compared to full-length Nbea (PH-BEACH: 3,606 ± 586.2, *P*_NbeaFL_ < 0.0001; **Figure 3D**). In addition, we compared the expression of these individual domains to full-length Nbea in the dendritic shaft which mimicked the expression in the spine head (**Figure 3E**). However, expression of Lectin, WD40 and DUF domains was more similar to PH-BEACH in the dendritic shaft (PH-BEACH: 6,122 ± 1,323, *N* = 11; Lectin: 3,505 ± 584, *N* = 12, *P*_PH-BEACH_ = 0.131; WD40: 4,823 ± 826, *N* = 12, *P*_PH-BEACH_ = 0.808; DUF: 4,859 ± 892, *N* = 11, *P*_PH-BEACH_ = 0.839; P values are from multiple comparison ANOVA). In contrast, Nbea FL and Armadillo domain showed low expression in dendritic shafts (Nbea FL: 1,181 ± 104, *N* = 19; Arm-Long: 1,592 ± 250, *N* = 12; **Figure 3E**). Together, these data indicate that all Nbea domains were reliably expressed in DSs, making their use in rescue assays meaningful. Moreover, the high levels of PH-BEACH in spine heads pointed to a particularly prominent role in regulating postsynaptic functions of excitatory synapses.

**FIGURE 4:**
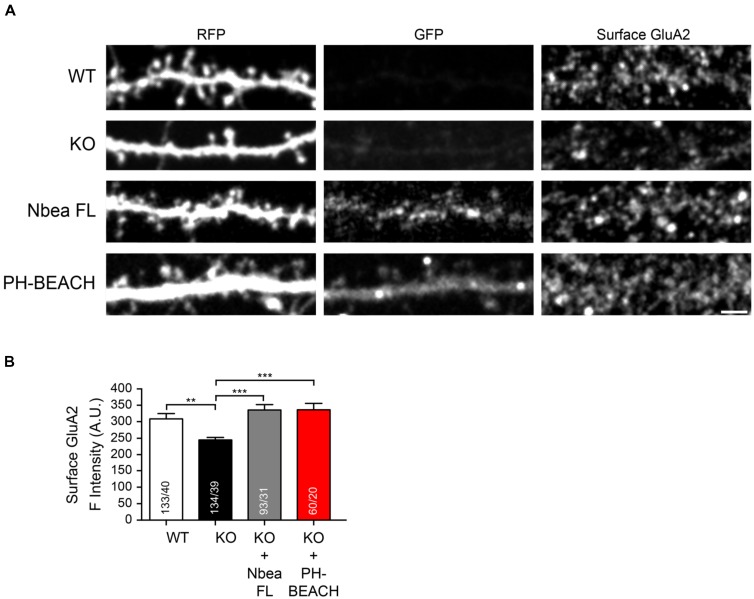
PH-BEACH domain restores surface targeting of GluA2 receptors in Nbea-deficient neurons. **(A)** Representative dendrites of primary hippocampal neurons from wild-type (WT) and Nbea null-mutant (KO) mice transfected with cytosolic marker t-dimer-RFP alone and in combination with GFP-tagged full-length Nbea (KO + Nbea FL) or the PH-BEACH domain (KO + PH-BEACH). Right panels, surface populations of GluA2 receptor subunits visualized by live labeling of neurons with an antibody directed against an extracellular epitope of the GluA2 subunit. Scale bar: 2.5 μm. **(B)** Quantification of surface GluA2 fluorescence intensity measured on dendrites of WT, KO, KO + Nbea FL and KO + PH-BEACH neurons. Data are means ± SEM. *N*, number of dendritic window/neurons (in bars); ^∗∗^*P* < 0.01, ^∗∗∗^*P* < 0.001, by one-way ANOVA with Tukey’s multiple comparisons test.

Since the phenotype of reduced amplitudes of EPSCs in Nbea KO neurons has been explained by an impaired surface targeting of AMPA receptor GluA2 subunits (Nair et al., 2013; Farzana et al., 2016), we asked if an individual domain of Nbea is able to re-establish normal dendritic GluA2 surface expression in KO neurons. To address this question, we performed live labeling of the surface population of endogenous GluA2 subunits in wild-type and Nbea null mutant neurons (**Figure 4A**). Nbea-deficient dendrites showed a diminished GluA2 surface staining compared to controls (WT: 309 ± 15 A.U., *N* = 133/40; KO: 244.3 ± 8.9, N = 134/39; *P* = 0.0036; **Figure 4B**), confirming an earlier report (Nair et al., 2013). The impairment could be fully rescued upon transfection of full-length Nbea in KO neurons (KO + Nbea FL: 335.6 ± 16.7 A.U., *N* = 93/31; *P* = 0.88; **Figures 4A,B**), attesting to the specificity of the phenotype. Most importantly, we found that expression of the PH-BEACH domain of Nbea alone in KO neurons was sufficient to restore GluA2 surface targeting back to control levels (KO + PH-BEACH: 336.5 ± 19.0 A.U., *N* = 60/20; *P* = 0.933; **Figures 3A,B**). These results identify the PH-BEACH complex of Nbea as the critical domain responsible for the impaired targeting of GluA2 receptors in KO neurons.

**FIGURE 5:**
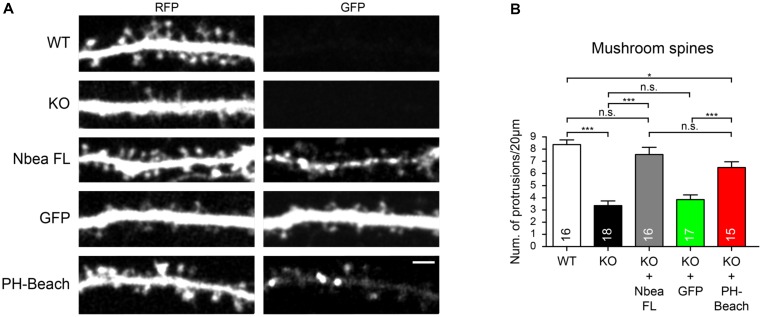
PH-BEACH domain of Nbea is sufficient to rescue the defect on mushroom spines. **(A)** Representative dendrites of primary hippocampal neurons from wild-type (WT) and Nbea null-mutant (KO) mice transfected with cytosolic marker t-dimer-RFP alone and in combination with GFP-tagged full-length Nbea (KO + Nbea FL) or PH-BEACH domain (KO + PH-BEACH). As additional control, KO neurons were mock transfected with GFP alone (KO + GFP). Scale bar: 2.5 μm. **(B)** Quantification of the number of mature mushroom-like DSs in different genotypes and upon expression of different constructs as indicated. Data are means ± SEM; *N*, number of neurons (in bars). Quantification was carried out analyzing the whole dendritic tree for each neuron. ^∗^*P* < 0.05 ^∗∗∗^*P* < 0.001, n.s., not significant by one-way ANOVA with Tukey’s multiple comparisons test.

We then asked if the PH-BEACH domain is also able to restore the normal number of DSs which was reduced in Nbea KO neurons (Niesmann et al., 2011) and which could possibly be linked to the GluA2 receptor defect (Nair et al., 2013; Farzana et al., 2016). To visualize the morphology of control and mutant hippocampal neurons, we transfected the cytosolic marker t-dimer-RFP (**Figure 5A**). Consistent with the earlier report (Niesmann et al., 2011), we determined a more than 50% reduction in the number of mature, mushroom-shaped DS in absence of Nbea (WT: 8.38 ± 0.38 protrusions/20 μm, *N* = 16; KO: 3.36 ± 0.38, *N* = 18; *P* < 0.001; **Figure 4B**). This impairment could be fully restored by expression of full-length Nbea in KO neurons (KO + NbeaFL: 7.56 ± 0.59 protrusions/20 μm, *N* = 16; *P*_WT_ = 0.693; **Figures 5A,B**). Strikingly, expression of the PH-BEACH domain alone was also able to double the number of DS compared to KO neurons (KO + PH-BEACH: 6.46 ± 0.46 protrusions/20 μm, *N* = 15; *P*_KO_ < 0.0001; **Figures 5A,B**), reaching numbers that came close to full-length Nbea (**Figure 5B**, *P*_KO+NbeaFL_ = 0.432). To exclude any off-target effects from the presence of a GFP epitope tag in our constructs, we additionally analyzed the spine numbers in Nbea KO neurons expressing a GFP without any Nbea sequence attached as control. No difference was found compared to KO neurons (KO + GFP: 3.85 ± 0.38 protrusions/20 μm, *N* = 17; *P*_KO_ = 0.926; **Figures 5A,B**), suggesting that our approach was reliable and all changes observed were due to the Nbea moiety. In contrast to mushroom spines, no significant difference of the number of stubby spines could be observed in WT, Nbea KO neurons, KO transfected with Nbea FL and KO transfected with GFP (*P* = 0.08; ANOVA). Thus, our results indicate that the PH-BEACH complex of Nbea is sufficient to restore normal numbers of mature mushroom-shaped DS in KO neurons.

### PKA Binding Site and Armadillo Domains Regulate Filopodia Formation

**FIGURE 6:**
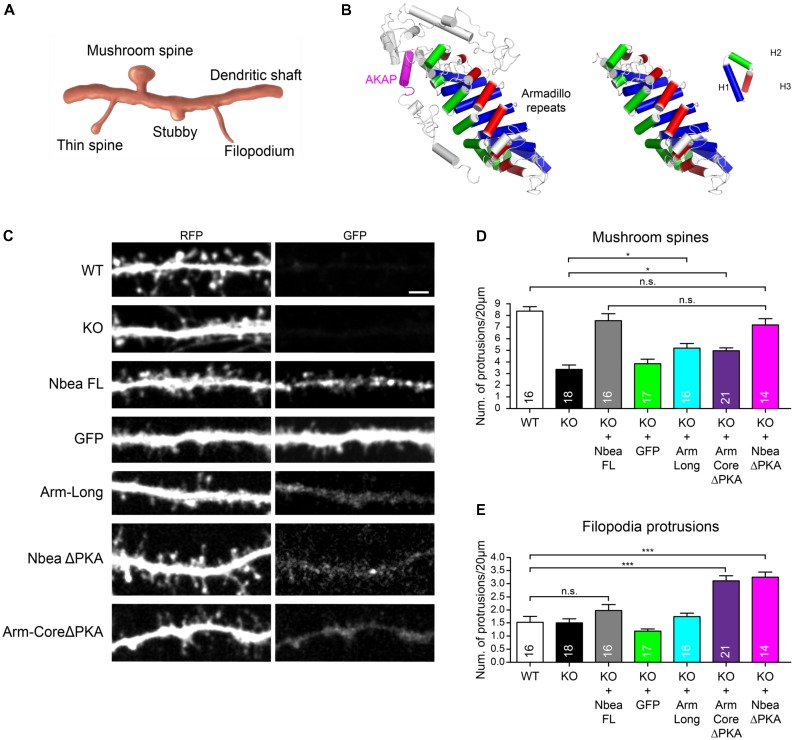
Armadillo domain and AKAP motif of Nbea affect filopodia formation. **(A)** Scheme depicting major categories of DS morphology, including mature mushroom spines, long thin spines, stubby spines, and filopodia. **(B)** Structural model of Arm-Long sequences (left) including the AKAP helix (magenta) and Arm-Core (right). An Armadillo domain consists of eight repeats of three helices H1 (blue), H2 (green), and H3 (red). The AKAP domain of Nbea is present in the Arm-Long construct but deleted in the Arm-Core ΔPKA construct **(C–E)**. **(C)** Representative dendrites of primary hippocampal neurons from wild-type (WT) and Nbea null-mutant (KO) mice transfected with cytosolic marker t-dimer-RFP alone and in combination with GFP-tagged full-length Nbea (KO + Nbea FL), Armadillo domain with AKAP motif (KO + Arm-Long), Armadillo domain without AKAP (KO + Arm-CoreΔPKA) and full-length Nbea lacking the AKAP (KO + NbeaΔPKA). As additional control, KO neurons were mock transfected with GFP alone (KO + GFP). Scale bar: 2.5 μm. **(D)** Quantification of number of mushroom-shaped DS in WT and Nbea KO neurons and upon expression of Nbea domains as detailed in **(C)**. Values for WT, KO, KO + FL, and KO + GFP are the same as in **Figure 4B** and displayed here again to allow for easy comparison with individual domains. Data are means ± SEM, *N*, number of neurons (in bars). Quantification was carried out by analyzing the whole dendritic tree for each t-dimer-RFP transfected neuron. ^∗^*P* < 0.05, n.s., not significant by one-way ANOVA with Tukey’s multiple comparisons test. **(E)** Quantification of filopodial protrusions in WT and Nbea KO neurons and upon expression of Nbea domains as detailed in **(C,D)**. Data are means ± SEM, *N*, number of neurons (in bars). Quantification was carried out analyzing the whole dendritic tree for each t-dimer-RFP transfected neuron. ^∗∗∗^*P* < 0.001, n.s., not significant by one-way ANOVA with Tukey’s multiple comparisons test.

The prominent effect of the PH-BEACH domain in restoring mushroom spine numbers shown above does not exclude, however, that other Nbea domains also contribute to the regulation of this or other classes of dendritic protrusions (**Figure 6A**). We first tested if the deletion of the PKA binding domain has an impact on DS by employing the NbeaΔPKA mutation that we used before to rescue the EPSCs (**Figure 1**). In line with our results obtained with the individual PH-BEACH domain (**Figures 4**, **5**), which is fully contained in the NbeaΔPKA construct and distant to the ΔPKA deletion site, the removal of AKAP did not affect the ability of Nbea to rescue mature DS numbers when transfected into KO neurons (NbeaΔPKA: 7.17 ± 0.56 protrusions/20 μm, *N* = 14; *P*_WT_ = 0.458 **Figures 6C,D**). However, we observed during the analysis of the dendritic tree of NbeaΔPKA transfected KO neurons (**Figure 6C**) that there were numerous thin and elongated protrusions called “filopodia” (**Figure 6A**). This is an interesting observation because larger numbers of filopodia occur during development and under conditions of reduced neural activity (Petrak et al., 2005). In our study, we classified every protrusion longer than 2 μm that lacked a well-defined head as “filopodium,” accepting that our class of filopodia might include some long spines but avoiding the problems associated with the resolution and orientation necessary to detect very small heads at the end of filopodial-like protrusions (Tonnesen et al., 2014). In fact, quantification of these protrusions showed that expression of NbeaΔPKA mutant more than doubled the number of filopodia in KO neurons (KO: 1.50 ± 0.16 protrusions/20 μm, *N* = 18, KO + NbeaΔPKA: 3.25 ± 0.19, *N* = 14; *P* < 0.0001; **Figure 6E**). These data may indicate that the PKA binding of Nbea could serve an unexpected role in negatively regulating the formation of filopodial-like dendritic protrusions.

PKA binding motifs fold into an amphipathic helix that binds a PKA RII dimer (Gold et al., 2013) and is freely accessible and not part of a larger domain (Smith et al., 2013). The helix anchors PKA, but the remaining domains of a particular AKAP define targeting to compartments and subsequent functions of that complex (Wong and Scott, 2004). In Nbea, an Armadillo domain resides next to the PKA RII binding site. To explore the effect on dendritic protrusions in more detail, we decided to compare the influence of a long Armadillo construct containing this PKA binding sequence (**Figure 6B** left, Arm-long) to an Armadillo “core domain” without PKA binding sequence (**Figure 6B** right, Arm-coreΔPKA). When expressed in Nbea KO neurons (**Figure 6C**), the Arm-long and the Arm-coreΔPKA domains had only weak effects on the number of mushroom DS (Arm-Long: 5.16 ± 0.39 protrusions/20 μm, *N* = 16; Arm-coreΔPKA: 4.95 ± 0.24, *N* = 21; *P* = 0.040 and *P*_KO_ = 0.062; **Figure 6D**). This confirms the importance of the PH-BEACH domain, not contained in the Arm constructs, to promote strongly formation of mature DS (**Figure 5**). However, we discovered that the Armadillo domain was able to increase the number of filopodia but only if the PKA binding site was missing. The Arm-coreΔPKA domain increased significantly the number of filopodia in Nbea-deficient neurons compared to all controls (Arm-coreΔPKA: 3.11 ± 0.19 protrusions/20 μm, *N* = 21; *P*_WT_ < 0.0001; **Figures 6C,E**), whereas the Arm-long domain failed to induce filopodia (Arm-long: 1.75 ± 0.13 protrusions/20 μm, *N* = 16; *P*_WT_ = 0.981; **Figure 6E**). Together, these results suggest that the Armadillo domain and PKA binding site of Nbea reciprocally regulate the number of filopodia on dendrites, with Armadillo showing a facilitating influence while the PKA site serves as a negative regulator.

### Actin Cytoskeletal Organization in Absence of Nbea

**FIGURE 7:**
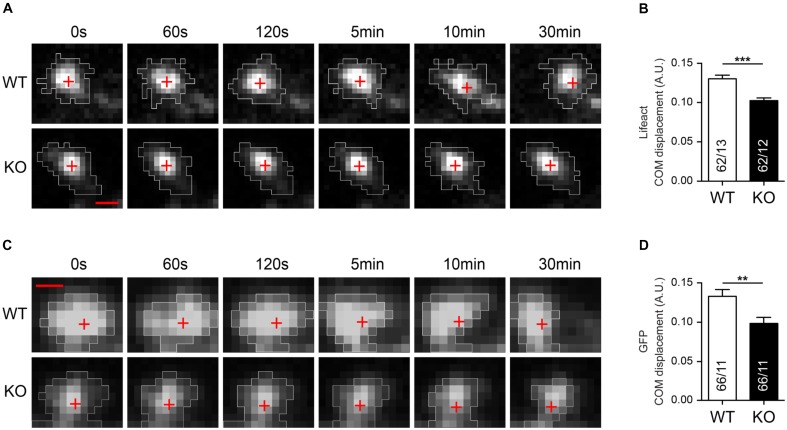
Impaired basal motility of Nbea-deficient DSs. **(A)** Representative image frames of WT and Nbea KO spines at different time points and transfected with Lifeact fused to RFP. The fluorescent signal of Lifeact-RFP highlights specifically polymerized filamentous F-Actin. White areas represent regions taken in consideration for center of mass (COM) displacement analysis in each time point. Red crosses depict the position of COM at each time point. **(B)** Quantification of COM displacement in WT and Nbea KO single spine. Data are means ± SEM, *N*, number of spines/neurons (in bars); ^∗∗∗^*P* < 0.001 by unpaired *t*-test. **(C,D)** Similar analysis to **(A,B)** using an alternative label of the spine volume, cytosolic GFP expressed in WT and Nbea KO neurons. White areas represent regions taken in consideration for COM displacement analysis in each time point. Red crosses depict the position of COM at each time point. Data are means ± SEM, *N*, number of spines/neurons (in bars); ^∗∗^*P* < 0.01 by unpaired *t*-test. Scale bar: 1 μm; pixel size = 0.211 μm.

Actin is the major cytoskeletal component of DSs and changes in the morphology of spines critically depend on the remodeling of the actin cytoskeleton (Frost et al., 2010; Hotulainen and Hoogenraad, 2010). Since DSs are an important substrate of activity-dependent dynamic remodeling in the brain (Hofer et al., 2009; Kwon and Sabatini, 2011), we decided to analyze if lack of Nbea affects the basal motility of DS. Hippocampal wild-type and Nbea-deficient neurons were transfected with Lifeact to specifically stain polymerized filamentous F-actin (Riedl et al., 2008) (**Figures 7A,B**) and with cytosolic GFP to visualize the entire volume of spines (**Figures 7C,D**). We then imaged spines for 30 min every 15 s and calculated the center of mass (COM) displacement over time for individual spines (**Figures 7A,C**). Our quantitative analysis revealed that deletion of Nbea caused both reduced F-actin related (WT: 0.128 ± 0.004, *N* = 62/13 A.U.; KO: 0.103 ± 0.003, *N* = 62/12; *P* < 0.0001; **Figure 7B**) and volume-based motility (WT: 0.133 ± 0.008, *N* = 66/11 A.U.; KO: 0.0985 ± 0.008, *N* = 66/11; *P* = 0.008; **Figure 7D**) of mutant spine heads compared to controls. These data suggest that the basal motility of DSs in KO mice is impaired, raising the question if and how the actin cytoskeleton might be regulated by Nbea.

**FIGURE 8:**
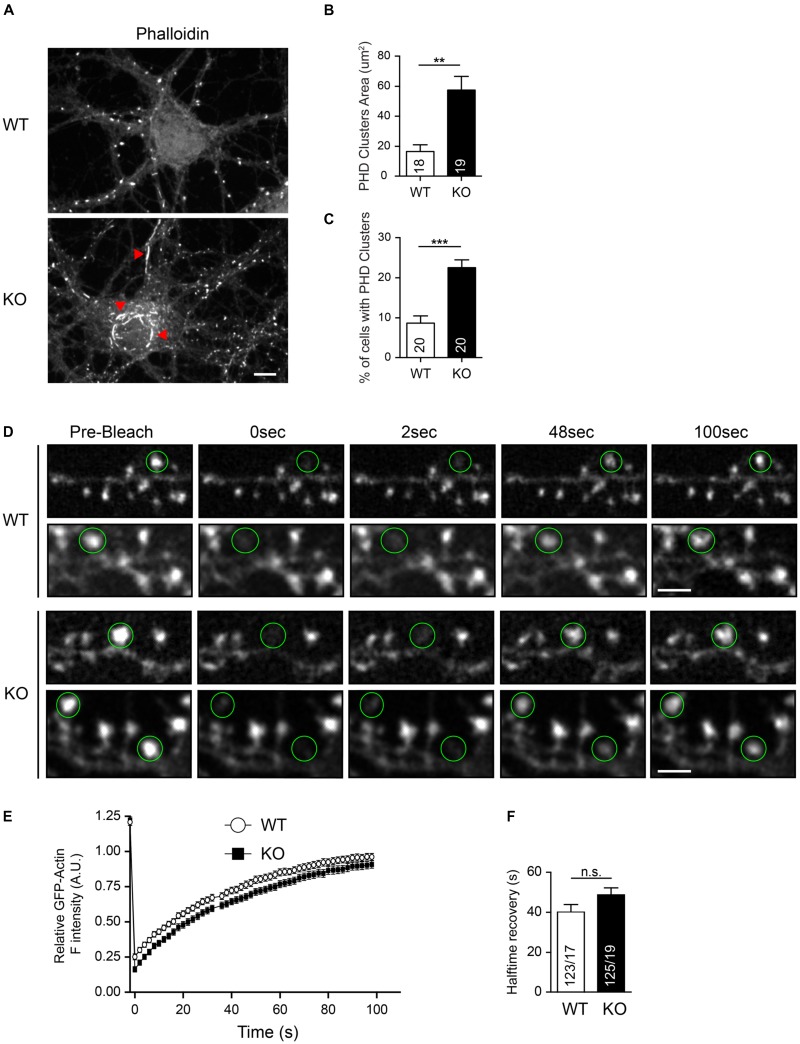
Normal actin polymerization and turnover in Nbea-deficient DSs. **(A)** Representative images of hippocampal WT and Nbea KO neurons stained with fluorescent phalloidin. Red arrowheads in images point to ectopic actin clusters mostly in the soma and soma-near large dendrites. Scale bar: 5 μm. **(B)** Quantification of actin cluster areas detected by phalloidin staining. Data are means ± SEM, *N*, number of neurons (in bars); ^∗∗∗^*P* < 0.001 by unpaired *t*-test. **(C)** Quantification of the percentage of cells with visible phalloidin-stained actin clusters in WT and KO cultures. Data are means ± SEM, *N*, number of visual fields counted (in bars); ^∗∗∗^*P* < 0.001 by unpaired *t*-test. **(D)** Representative images of FRAP experiments of wild-type (WT) and Nbea-deficient (KO) spines at different time points. Samples show GFP-actin transfected dendrites at the indicated time points after bleaching, pre-Bleach indicates spines before quenching. Green circles, region selected for bleaching around spine heads. Scale bar: 2.5 μm. **(E,F)** Quantification of FRAP data with first order exponential equation fitting of relative fluorescence intensity of GFP-Actin values over time **(E)**. Halftime recovery of GFP-Actin was derived from exponential curves **(F)**. Data are means ± SEM, *N*, number of spines/neurons (in bars); n.s., not significant by unpaired *t*-test.

In support of the importance of the actin cytoskeleton, we previously obtained indirect evidence that the actin organization may be involved in the spine phenotype of Nbea KO neurons (Niesmann et al., 2011). We now built on this study and demonstrate that KO neurons contain increased areas of phalloidin-labeled clusters compared to WT (WT: 16.44 ± 4.61 μm^2^, *N* = 18; KO: 57.68 ± μm^2^, *N* = 19; *P* < 0.01; **Figures 8A,B**). Moreover, Nbea KO cultures contained an increased percentage of neurons with such phalloidin-stained actin clusters that were mostly found ectopically in the soma (WT: 8.66 ± 1.84%, *N* = 20; KO: 22.68 ± 2.02, *N* = 20; *P* < 0.001; **Figure 8C**). Our findings on DS numbers, filopodia formation and basal spine motility raised the question if Nbea acts directly on the actin cytoskeleton, for example, by modulating actin polymerization rates in DS. To analyze if changes in actin polymerization rates were responsible for the spine phenotype, we performed FRAP experiments on single spines by expressing GFP-actin in wild-type and Nbea-deficient neurons (**Figure 8D**). However, KO spines showed no differences in fluorescence intensity recovery curves compared to controls (**Figure 8E**). Moreover, an analysis of the halftime of GFP-actin fluorescence recovery of WT and Nbea KO DS revealed only a tendency, albeit not significant, toward longer recovery halftime (WT: 20.16 ± 1.79 s, *N* = 123/17; KO: 24.38 ± 1.72, *N* = 125/19; *P* = 0.097; **Figure 8F**). These results indicate that Nbea does not, or at least not prominently, regulate the actin polymerization dynamics in DS. Future research will have to test other mechanisms, for example related to branching of the dendritic actin cytoskeleton, that may mediate the important effects of Nbea on spinous synapses.

## Discussion

Here, we report that expression of full-length human Nbea is able to restore normal excitatory postsynaptic currents in neurons lacking endogenous Nbea. We demonstrate that an isolated motif of the multidomain protein Nbea, PH-BEACH, is sufficient to correct one deficiency of Nbea KO neurons, the impaired surface targeting of GluA2 AMPA receptors. Moreover, both full-length Nbea and PH-BEACH alone are able to restore a normal density of mature mushroom spine numbers at excitatory synapses, whereas the PKA RII binding site and the Armadillo domain of Nbea reciprocally regulate filopodial extension, a transient class of dendritic protrusions.

### PH-BEACH Domain Mediates Key Functions of Nbea

Our study aimed at the identification of functions that are specific for individual domains of Nbea, a membrane-associated protein classified by its BEACH domain. Crystallization of BEACH domains remained elusive until the sequence was extended by 130 residues to the N-terminus (Jogl et al., 2002). This additional sequence was an untypical PH domain, revealing a unique insertion of two helices into the canonical fold motif. Furthermore, it lacks a lipid binding loop consisting of basic residues that coordinate phosphate ions of phosphatidylinositol phosphates (PIP) in other PH domains as in GRP1 (PDB_ID: 1FHW, Ferguson et al., 2000). Rather, the loop in PH of Nbea is made of hydrophobic residues that form a hydrophobic cluster with the BEACH domain (Jogl et al., 2002; Gebauer et al., 2004). A surface plasmon resonance binding assay of isolated recombinant domains confirmed their tight complex and determined the PH-BEACH domain tandem as a structural unit (Gebauer et al., 2004). Consistent with the missing phosphate binding loop, PIP binding was not immediately detectable for PH-BEACH domains of Nbea and LRBA (Jogl et al., 2002; Gebauer et al., 2004) but was present for FAN, another member of the family that consists solely of PH-BEACH and WD domains. The basic residues identified for PIP binding of FAN are located untypically at the surface of the PH domain (Haubert et al., 2007) and are not conserved in Nbea or LRBA. This feature is reminiscent of a similar tight PH-phosphotyrosine phosphatase (PTP) tandem, where a phosphoinositol phosphate binds to the PTP domain, but not to the PH (Begley et al., 2006). These authors concluded based on the structure that the PH domain has contact to PIPs, albeit with low affinity. By analogy, the PH-BEACH domains of Nbea and LRBA, despite forming tight structural unit, might still attach to PIPs as they contain basic surface regions. This putative low affinity PIP interaction has to await experimental confirmation but could be relevant to the effect of the PH-BEACH domain on GluA2 targeting to the postsynaptic membrane. This is an important question since no other binding partners have been identified yet for the PH-BEACH domain within Nbea or in any other PH-BEACH containing molecule (Cullinane et al., 2013).

More structural insight came from a study showing the C-terminal half of Nbea binds to SAP102, a MAGUK molecule involved in trafficking of ionotropic receptors (Lauks et al., 2012). Although the PH-BEACH domain is contained in the C-terminal half along with the DUF and WD domains (compare **Figure 3A**), the isolated PH-BEACH domain failed to bind to SAP102 (Lauks et al., 2012). Moreover, a point mutation introduced into the PH-BEACH domain (E2218R) of their C-terminal Nbea construct, which possibly disrupted the tight structural unit between PH and BEACH (Jogl et al., 2002), also prevented SAP102 binding. These data suggest that the PH-BEACH domain itself does not contain a SAP102 binding site but that the integrity of PH-BEACH determines the conformation of the entire C-terminus. To facilitate future experimental work, we therefore propose a structural model of full-length Nbea (**Figure 3B**) that is based on a compact C-terminus in which the PH-BEACH domain interacts with the flanking DUF and WD domains. In support, there is another WD repeat upstream of the DUF that can be identified by sequence homology (residues 1326–1368, UniProt). This repeat might form a 5-bladed WD propeller fold together with the four WD repeats (WD40) at the C-terminus (green in **Figure 3B**) that should clamp the C-terminus even tighter into a compact domain arrangement.

In spite of the predicted compact structure of the C-terminus of Nbea, we observed that the PH-BEACH can be functional as an individual unit: it localized prominently to the spine head (**Figure 3**), rescued the surface expression of GluR2 AMPA receptors (**Figure 4**) and restored normal numbers of mature mushroom spines (**Figure 5**). Consistent with a prominent role at excitatory spine synapses, the E2218R mutation in PH-BEACH of full-length Nbea impaired excitatory but not inhibitory neurotransmission (Farzana et al., 2016). While this defect can be interpreted by the loss of SAP102 binding (Farzana et al., 2016), our results emphasize an alternative role of PH-BEACH at the postsynaptic density that is independent of SAP102. The latter is corroborated by the fact that glutamate receptor signaling was unchanged in SAP102 null mutant neurons (Farzana et al., 2016).

### Role of PKA Binding Site in Nbea

Our rescue experiments with the isolated PH-BEACH domain implicated that its role is independent of the PKA RII binding site of Nbea, a hallmark of the protein (Wang et al., 2000). Consistently, we found that deletion of the binding site (NbeaΔPKA) does not abolish the ability of full-length Nbea to rescue most properties of EPSCs (**Figures 1**, **2**) or mushroom spine numbers (**Figure 5**). At the same time, we discovered a new role for the PKA RII binding site: we observed increased filopodia formation in its absence (shown by NbeaΔPKA and ARM-coreΔPKA constructs), whereas full-length Nbea and the armadillo domain with PKA RII binding site behaved normally (**Figure 5**). It can be concluded that binding of PKA to Nbea prevents or limits filopodia formation which by default might be promoted by the armadillo domain. In fact, PKA dependent processes have been shown to facilitate filopodia formation in other studies (Chen et al., 2003; Lin et al., 2007). Similarly, other armadillo domains, for example from δ-catenin, are also involved in filopodia formation (Abu-Elneel et al., 2008). Since many AKAPs are integrated in protein networks involving cdc42 (Poelmans et al., 2013), Nbea might regulate the protrusive activity for filopodia extension through the cdc42-cofilin-actin pathway.

Our electrophysiological analysis of the NbeaΔPKA mutant revealed another relevant aspect of the impaired regulation of filopodia because we observed that NbeaΔPKA restores mEPSCs rise time and inter-event interval but not decay time. Recent work correlated the imbalanced number of filopodia protrusions with EPSCs impairments: for example, knock-down of class II Myosin motor heavy chain *MyH7B* in rat hippocampal neurons causes irregularly shaped DS heads with filopodia protrusions and impaired mEPSCs (Rubio et al., 2011). Conversely, neurons depleted of the 4E-binding-protein-2 (4E-BP2), a repressor of mRNA translation, displayed lower number of filopodia protrusion and increased evoked and miniature EPSCs (Ran et al., 2013). The higher number of filopodia seen upon expression of NbeaΔPKA in Nbea KO neurons could therefore explain its inability to rescue decay time of mEPSCs due to the absence of a functional postsynaptic density in filopodia protrusions.

The question of additional targets of the PKA signaling of Nbea remains open. The PKA signaling pathway in DSs is known, for example, to lead to reduced phosphorylation of GluA1 as was shown for the melanocortin GPCR receptor. MC4R stimulates surface GluA1 trafficking through phosphorylation at Ser845 in a Gα-cAMP/PKA-dependent manner (Shen et al., 2013). Possibly, a similar mechanism may affect the serine phosphorylation of GluA2 subunits which were reduced in surface trafficking in Nbea KO neurons. While experimental proof for such a scenario is lacking, at least PKC is able to phosphorylate GluA2 at serine 880 and PKA phosphorylation of GluA1 induces exocytosis to extrasynaptic sites and primes AMPA receptors for CamKII dependent synaptic delivery (Henley et al., 2011). Although deletion of Nbea revealed a particularly strong reduction of GluA2 subunit at the surface (**Figure 4** and Nair et al., 2013), diminished surface levels of GluA1 and GluK2/3 kainate receptors have also been described (Nair et al., 2013). Thus, the PKA binding site of Nbea might be critical for the fine-tuning of the phosphorylation status of several AMPA receptor subunits, collectively leading to the impaired mEPSCs decay time and imbalance of different classes of DSs.

Finally, the PKA signaling of Nbea could also involve synaptopodin, a key molecule of the spine apparatus (Deller et al., 2003), which is ectopically retained in the *trans*-Golgi network together with actin clusters in Nbea KO neurons (Niesmann et al., 2011). Synaptopodin has recently been described as PKA substrate essential for the regulation of NMDA receptor-dependent synaptic plasticity and spine expansion upon chemical LTP induction (Faul et al., 2008; Zhang et al., 2013). Thus, Nbea could recruit a molecular complex regulating synaptopodin phosphorylation through its interaction with PKA RIIα subunit, and thereby inducing actin cytoskeleton remodeling. In presence of our NbeaΔPKA mutant that is unable to interact with PKA, synaptopodin regulation could be lost or impaired, leading to defective actin cytoskeleton remodeling and filopodia formation. On the other hand, if Nbea influences via its interaction with PKA the amount of active NMDA channels in the postsynapse, then the lack of rescue of the mEPSC decay time by NbeaΔPKA may reflect the inability of this truncated form to support the slower NMDA-component of the postsynaptic current. Although our results showed that the actin polymerization and turnover rate are normal in Nbea KO neurons, the impairment of the actin cytoskeleton in DSs could involve other aspects such as actin branching or capping, which future research will have to address.

## Author Contributions

DR, JB, CL, LN, WG, HR, and CR generated the data. DR, JB, JR, CR, and MM analyzed the data. MK provided mutant mice. DR, JR, and MM designed the study. DR and MM wrote the manuscript. All authors contributed to manuscript revision, read, and approved the submitted version.

## Conflict of Interest Statement

The authors declare that the research was conducted in the absence of any commercial or financial relationships that could be construed as a potential conflict of interest.
